# The Association of Early Graft Dysfunction and CYP3A5 Genotype With Tacrolimus Exposure in Adult Kidney Transplant Recipients

**DOI:** 10.1002/phar.70216

**Published:** 2026-11-09

**Authors:** Meghan Schuff Zimmerman, Amy L. Pasternak, Snehal Patil, Sebastian Zöllner, Jeong M. Park

**Affiliations:** ^1^ Department of Pharmacy Michigan Medicine Ann Arbor Michigan USA; ^2^ Department of Clinical Pharmacy University of Michigan College of Pharmacy Ann Arbor Michigan USA; ^3^ Department of Biostatistics University of Michigan School of Public Health Ann Arbor Michigan USA

**Keywords:** CYP3A5, delayed graft function, kidney transplant, tacrolimus

## Abstract

**Introduction:**

The incidence of early graft dysfunction following kidney transplantation is rising due to the expanded use of marginal organs. Prior studies have suggested that acute kidney dysfunction causes renohepatic crosstalk and tacrolimus metabolism may be decreased. It is also well known that tacrolimus metabolism and dosing requirements differ significantly based on a recipient's *CYP3A5* genotype. The objective of this study was to investigate the interplay between early graft function and pharmacogenetics on tacrolimus dose requirements in adult kidney transplant recipients.

**Methods:**

This was a single‐center, retrospective cohort study of adult kidney transplant recipients who received immediate‐release tacrolimus and had *CYP3A5* genotypes available. Immediate graft function (IGF) was defined as serum creatinine decrease of ≥ 10% from baseline and urine output ≥ 0.5 mL/kg/h on post‐operative day (POD) 1. Tacrolimus dose‐normalized trough concentration (C/D) on POD 2 was compared between patients with IGF and non‐IGF using Mann–Whitney U test. Patients were then grouped by both IGF and *CYP3A5* genotype, and the differences in C/D considering these factors were analyzed by Mann Whitney U test and a multivariable linear regression analysis.

**Results:**

A total of 242 patients were included, 185 with IGF and 57 with non‐IGF. The median [IQR: interquartile range] of tacrolimus C/D was higher in non‐IGF than IGF (200.0 [129.7–327.1] vs. 134.0 [95.0–197.4] [ng/ml]/[mg/kg/day], *p* < 0.001). Among CYP3A5 nonexpressers, the median [IQR] of tacrolimus C/D was 73% higher in non‐IGF than IGF (263.3 [177.5–390.1] vs. 151.8 [105.1–214.1] [ng/ml]/[mg/kg/day], *p* < 0.001), and 16% higher in CYP3A5 expressers with non‐IGF versus IGF (116.2 [102.3–199.5] vs. 101.1 [72.5–131.0] [ng/ml]/[mg/kg/day], *p* = 0.01). In the multivariable linear regression model, the effects of IGF and CYP3A5 expression on tacrolimus C/D remained significant.

**Conclusion:**

Patients with early graft dysfunction who were CYP3A5 nonexpressers had the highest tacrolimus exposure. Initial tacrolimus dose reductions may be considered in kidney transplant recipients who are at risk for non‐IGF, especially in CYP3A5 nonexpressers.

## 1 Introduction

Tacrolimus is the backbone of the maintenance immunosuppression regimen used in kidney transplantation. It has a narrow therapeutic index and requires careful monitoring and management. High pharmacokinetic variability between patients makes the balance between the desired therapeutic effects of immunosuppression and toxicities such as nephrotoxicity difficult to manage [1]. Interpatient variability in tacrolimus pharmacokinetics is attributed primarily to cytochrome P450 (CYP) 3A5 genetic polymorphisms. Patients who have at least one copy of the wildtype CYP3A5*1 allele express the CYP3A5 enzyme. When compared to CYP3A5 nonexpressers (i.e., those who do not have a CYP3A5*1 allele), CYP3A5 expressers have lower, often subtherapeutic, tacrolimus trough concentrations when given a standard starting dose [2, 3].

In recent years, the kidney transplant donor pool in the United States has expanded to use marginal organs that were previously considered high‐risk for graft dysfunction [4]. This includes grafts from donors with higher body mass indexes, hypertension, diabetes, and longer cold ischemic times. Although this approach led to shorter wait times and higher rates of kidney transplant, the function of grafts from high‐risk donors may be compromised immediately following transplant [4, 5]. A key part of managing immunosuppression in the early post‐transplant period should consider altered pharmacokinetics in the setting of early graft dysfunction to minimize adverse effects, such as nephrotoxicity and rejection.

Several previously published studies indicate that acute kidney dysfunction may alter CYP3A metabolism via renohepatic crosstalk [6, 7]. In a study using midazolam as in vivo surrogates of CYP3A4 and CYP3A5 activity, a significant increase in midazolam concentrations occurred in patients with acute kidney injury (AKI). This effect was further enhanced in patients who were CYP3A5 nonexpressers, which indicated that not only does renal function affect hepatic metabolism through CYP3A4/3A5, but such effects may be attenuated by CYP3A5 expression [7]. A study in kidney transplant recipients reported that patients who experienced post‐operative delayed graft function (DGF) had higher tacrolimus levels [8]. This is concerning because supratherapeutic tacrolimus levels may lead to worsening graft function in the setting of early graft dysfunction. It may also lead to further kidney injury in grafts with previous insult and slower recovery from DGF. Therefore, determining the interplay of early graft dysfunction and CYP3A5 expression on tacrolimus exposure is imperative for optimal immunosuppression post‐kidney transplant. By customizing tacrolimus dosing based on graft dysfunction, we may prevent further injury to grafts at risk for DGF.

This study aimed to assess whether early graft function influences tacrolimus concentrations in adult kidney transplant recipients, and whether the impact of early graft function varies among CYP3A5 expressers and nonexpressers. We hypothesized that both early graft dysfunction and CYP3A5 expression would impact the dose‐normalized tacrolimus exposure (C/D) and that patients who experience early graft dysfunction and are CYP3A5 nonexpressers would have the highest tacrolimus C/D, indicating lower dose requirements.

## 2 Methods

This single‐center, retrospective, cohort study was conducted at Michigan Medicine. This study was reviewed and approved by the University of Michigan institutional review board (HUM00143486; HUM00161844).

### 2.1 Study Patients

Patients ≥ 18 years old who received a kidney transplant at Michigan Medicine between July 1, 2014 and September 31, 2024, initiated immediate‐release oral tacrolimus (IR‐Tac) as part of their immunosuppression, had clinical tacrolimus troughs collected on post‐operative day (POD) 2 after receiving three consecutive unchanged doses of IR‐Tac, and had a clinical or research *CYP3A5* genotype result available were included in this study.

Patients who received a previous liver transplant, underwent multiple organ transplants, had tacrolimus exposure prior to transplant, or participated in research studies at the time of transplant were excluded. Patients who took medications known to have strong drug–drug interactions with tacrolimus were also excluded. Medications considered to have strong drug–drug interactions with tacrolimus included azole antifungals, protease inhibitors, amiodarone, diltiazem, verapamil, macrolide antibiotics (except for azithromycin), phenytoin, phenobarbital, oxcarbazepine, rifampin, and carbamazepine. Patients taking sublingual, extended‐release, or intravenous formulations of tacrolimus, or who received varying doses of IR‐Tac prior to their POD 2 trough level were excluded.

Included patients were categorized by early graft function. Immediate graft function (IGF) was defined as serum creatinine (SCr) reduction of ≥ 10% from the pre‐transplant baseline and urine output ≥ 0.5 mL/kg/h on POD 1. Patients who did not meet the criteria for IGF or who required renal replacement therapy within the first 7 days following transplantation were classified as having non‐immediate graft function (non‐IGF). In this study, non‐IGF was intentionally defined more broadly than the conventional definition of DGF, which is typically limited to dialysis requirement within 7 days of transplantation, to capture clinically relevant early graft dysfunction that may not necessitate dialysis. This approach was selected to better align with the study objective of evaluating the association between early graft dysfunction and tacrolimus exposure.

Patients were also categorized according to their CYP3A5 phenotype. Patients carrying at least one CYP3A5*1 allele were categorized as CYP3A5 expressers. All other *CYP3A5* genotypes were categorized as nonexpressers [2].

### 2.2 Data Collection

The electronic health record (EHR) was utilized for secure data collection. All collected data were securely stored in RedCap [9, 10]. *CYP3A5* genotype was collected from either the Michigan Genomics Initiative Biorepository or the EHR if testing had been performed for clinical use [11]. *CYP3A5* genotypes from either resource included assessment for CYP3A5*3, CYP3A5*6, and CYP3A5*7 alleles; a CYP3A5*1 allele was inferred on each gene copy where these variants were not detected. Data collection included tacrolimus dosing, tacrolimus whole blood trough concentrations, and allograft function through POD 7. Demographics including age, race, sex, pre‐operative weight, end stage renal disease etiology of the recipients, donor type, and concomitant medications were also collected.

### 2.3 Immunosuppression Protocol and Tacrolimus Dosing

According to institutional protocol, standard maintenance immunosuppression consisted of tacrolimus, mycophenolate, and prednisone. Rabbit antithymocyte globulin (rATG) induction was indicated for patients with immunological risk factors or for those receiving steroid‐avoidant maintenance immunosuppression.

The initial dosing of IR‐Tac was standardized to 0.075 mg/kg administered orally every 12 h prior to the implementation of *CYP3A5* genotype dosing in October 2023. After October 2023, patients underwent *CYP3A5* genotyping as part of their transplant evaluation and the recommended IR‐Tac dosing was based on their CYP3A5 phenotype. CYP3A5 nonexpressers had a recommended starting dose of 0.075 mg/kg/dose, intermediate metabolizers (carriers of one CYP3A5*1 allele) had a recommended starting dose of 0.1 mg/kg/dose, and normal metabolizers (carriers of two CYP3A5*1 alleles) had a recommended starting dose of 0.15 mg/kg/dose [12]. The dose was calculated using actual body weight, and the first dose was administered within 24 h of transplantation at the first scheduled dosing time (9 a.m. or 9 p.m.).

For the primary outcome, tacrolimus dose‐normalized trough concentration (C/D; [ng/ml]/[mg/kg/day]) on POD 2 was calculated for each patient using the weight‐normalized total daily dose administered before trough sampling. The first tacrolimus trough concentration was routinely obtained on POD 2, after which tacrolimus dose adjustments were often made based on the measured level. Therefore, POD2 was selected to evaluate tacrolimus exposure before any dose modifications. To further standardize the analysis, only patients who had received three consecutive unchanged doses of IR‐Tac before the POD 2 trough measurement were included.

### 2.4 Statistical Analysis

Demographics were compared between patients with IGF and non‐IGF. Categorical variables were assessed using chi‐square tests and expressed as number and percentage. Continuous variables were assessed using nonparametric tests (Mann–Whitney U) and expressed as median and interquartile range (IQR). The difference in the tacrolimus C/D between the IGF and non‐IGF groups was assessed using the Mann–Whitney U test. Patients were then grouped by CYP3A5 expression and graft function, and the tacrolimus C/D was compared between the IGF and non‐IGF groups within the same CYP3A5 phenotype. The impact of early graft function and CYP3A5 expression on the tacrolimus C/D was also assessed using a multivariable linear regression controlling for age, race, donor type, induction (rATG vs. none), and maintenance immunosuppression (standard vs. steroid avoidant). The Ln(tacrolimus C/D) was used in the regression given nonnormal distribution. A 2‐sided *p*‐value of < 0.05 was considered statistically significant.

## 3 Results

A total of 352 kidney transplant recipients with *CYP3A5* genotype results were screened. Of those, patients were excluded due to varying tacrolimus doses prior to the POD 2 trough (*n* = 74), missing demographic data (*n* = 13), use of tacrolimus formulations other than IR‐Tac (*n* = 12), prior tacrolimus use (*n* = 9), and participation in a research study at the time of transplant (*n* = 2). This left 242 transplant recipients included in our data analysis. Of those, 185 (76.4%) patients had IGF and 57 (23.6%) patients had non‐IGF following transplant. When considering early graft function and CYP3A5 expression combined, 43 (17.8%) patients were IGF/CYP3A5 expressers, 16 (6.6%) were non‐IGF/CYP3A5 expressers, 142 (58.7%) were IGF/CYP3A5 nonexpressers, and 41 (16.9%) were non‐IGF/CYP3A5 nonexpressers (Figure 1).

**FIGURE 1:**
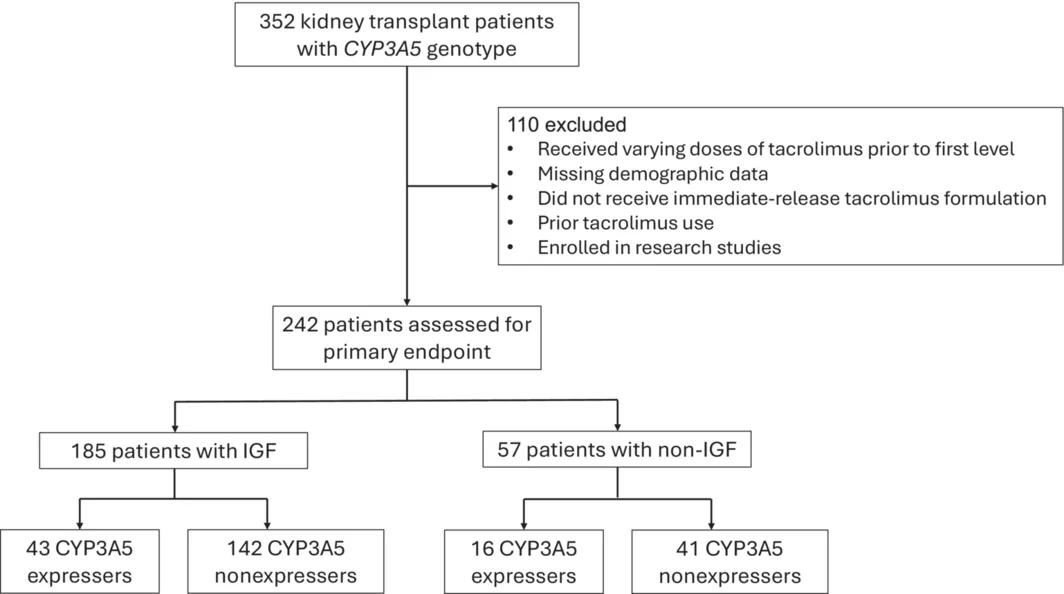
Consort diagram of patient inclusion. CYP, cytochrome P450; IGF, immediate graft function.

Table 1 shows the baseline characteristics between the IGF and non‐IGF groups. The groups differed in age, race, donor type, pre‐operative weight, and induction and maintenance immunosuppression. In the non‐IGF group, patients were older at the time of transplant, had a higher pre‐operative weight, and were more likely to receive a kidney from deceased donors. A higher percentage of non‐IGF patients received rATG induction and standard steroid maintenance immunosuppression. In the IGF group, a higher percentage of patients were white and received a kidney from a living donor.

**TABLE 1:** Baseline characteristics.

Parameter	IGF, (*n* = 185)	Non‐IGF, (*n* = 57)	*p*
Age at transplant, (y), median (IQR)	52.3 (39.6–62.8)	56.8 (46.9–65.8)	0.03
Race, no (%)			0.02
White	139 (75.1)	34 (59.6)	
Black or African American	24 (13.0)	19 (33.3)	
Asian	5 (2.7)	1 (1.8)	
Native Hawaiian or other Pacific Islander	1 (0.5)	0 (0)	
Other/unknown	16 (8.6)	3 (5.3)	
Sex, no (%)			0.72
Male	112 (60.5)	33 (57.9)	
Female	73 (39.5)	24 (42.1)	
Ethnicity, no (%)			0.53
Non‐Hispanic or Latino	171 (92.4)	55 (96.5)	
Hispanic or Latino	13 (7.0)	2 (3.5)	
Other or unknown	1 (0.5)	0 (0)	
ESRD etiology, no (%)			0.288
Diabetes mellitus (Type 1 and 2)	56 (30.3)	24 (42.1)	
Hypertensive nephrosclerosis	23 (12.4)	9 (15.8)	
Focal segmental glomerular sclerosis	11 (5.9)	0 (0.0)	
Polycystic kidney disease	15 (8.1)	6 (10.5)	
IgA nephropathy	18 (9.7)	2 (3.5)	
Systemic lupus erythematosus	3 (1.6)	2 (3.5)	
Other	59 (31.9)	14 (24.6)	
Donor type, no (%)			< 0.001
Living Donor	127 (68.6)	5 (8.8)	
DBD	43 (23.2)	31 (54.4)	
DCD	15 (8.1)	21 (36.8)	
Pre‐operative weight (kg), median (IQR)	77.1 (68.0–93.8)	93.0 (75.2–108.6)	< 0.001
Induction immunosuppression, no (%)			< 0.001
rATG induction	123 (66.5)	52 (91.2)	
None	62 (33.5)	5 (8.8)	
Maintenance immunosuppression, no (%)			0.02
Standard	163 (88.1)	56 (98.2)	
Steroid avoidant	22 (11.9)	1 (1.8)	

Abbreviations: DBD, donation after brain death; DCD, donation after cardiac death; ESRD, end stage renal disease; IGF, Immediate graft function; IQR, interquartile range; POD, post‐operative day; rATG, rabbit antithymocyte globulin.

The tacrolimus C/D on POD 2 was significantly higher in the non‐IGF group compared to the IGF group (median [IQR]: 200.0 [129.7–327.1] vs. 134.0 [95.0–197.1] [ng/ml]/[mg/kg/day], *p* < 0.001) (Figure 2). Among the four early graft function/CYP3A5 expression subgroups, the tacrolimus C/D was highest in CYP3A5 nonexpressers with non‐IGF (Figure 3). CYP3A5 nonexpressers who developed non‐IGF had a 73% higher tacrolimus C/D compared to IGF (263.3 [177.5–390.1] vs. 151.8 [105.1–214.1] [ng/ml]/[mg/kg/day], *p* < 0.001). In CYP3A5 expressers, the difference in the tacrolimus C/D between patients was smaller, with 16% higher C/D in patients with non‐IGF (116.2 [102.3–199.5] vs. 101.1 [72.5–131.0] [ng/ml]/[mg/kg/day], *p* = 0.01).

**FIGURE 2:**
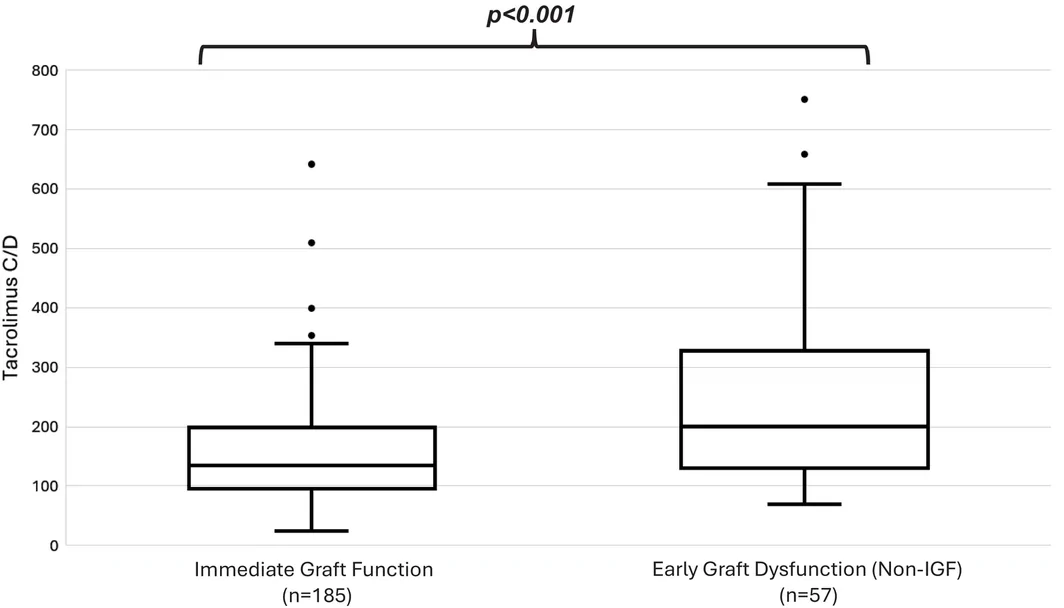
Tacrolimus dose‐normalized trough concentration on POD 2 in kidney transplant patients with IGF and non‐IGF. The tacrolimus C/D on POD 2 was significantly higher in non‐IGF patients compared to those with IGF (median [IQR]: 200.0 [129.7–327.1] vs. 134.0 [95.0–197.4] [ng/ml]/[mg/kg/day], *p* < 0.001). C/D, dose‐normalized trough concentration; IGF, immediate graft function; IQR, interquartile range; POD, post‐operative day.

**FIGURE 3:**
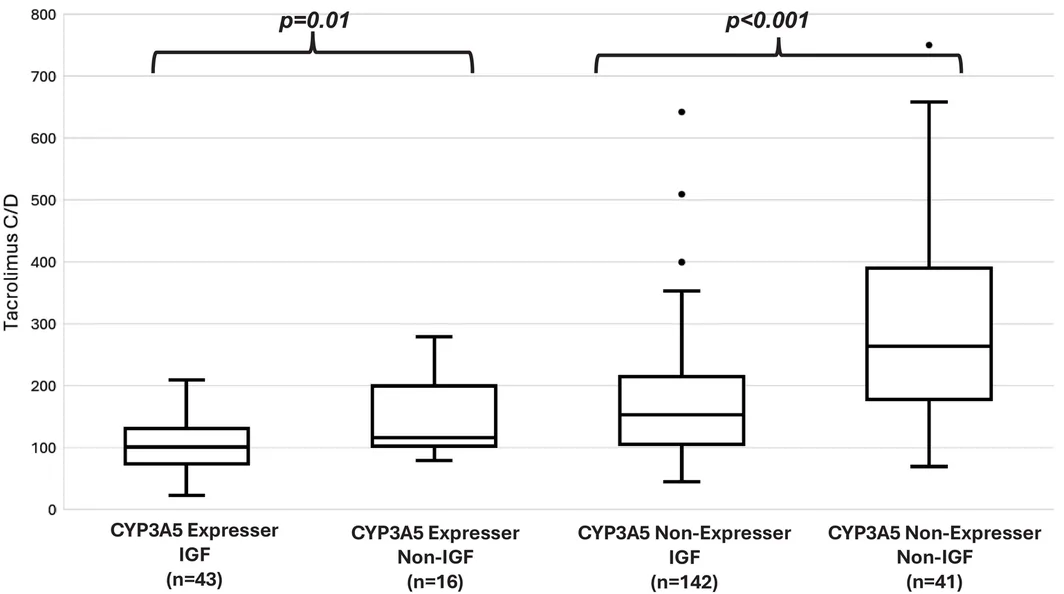
Tacrolimus dose‐normalized trough concentration on POD 2 in CYP3A5 expressers and nonexpressers with IGF and non‐IGF. CYP3A5 nonexpressers with non‐IGF had higher tacrolimus C/D compared to those with IGF (median [IQR]: 263.3 [177.5–390.1] vs. 151.8 [105.1–214.1] [ng/ml]/[mg/kg/day], *p* < 0.001); the tacrolimus C/D was also higher in CYP3A5 expressers with non‐IGF compared to those with IGF (median [IQR]: 116.2 [102.3–199.5] vs. 101.1 [72.5–131.0] [ng/ml]/[mg/kg/day], *p* = 0.01). C/D, dose‐normalized trough concentration; CYP, cytochrome P450; IGF, immediate graft function; IQR, interquartile range; POD, post‐operative day.

In the multivariable linear regression model, the effects of early graft dysfunction and CYP3A5 expression on the tacrolimus C/D remained significant after controlling for other covariates (Table 2). Of significance, patients with IGF had a 54% lower tacrolimus C/D compared to those with non‐IGF. CYP3A5 expressers had a 70% lower tacrolimus C/D than nonexpressers. In addition to IGF and CYP3A5 expression, age was also significantly associated with the tacrolimus C/D. For each year the patient's age increases, their tacrolimus exposure is predicted to increase by 1%.

**TABLE 2:** Multivariable linear regression of the Ln(Tacrolimus C/D).

Variables	Coefficient	95% CI	*t*	*p*
Black race	−0.107	−0.313, 0.099	−1.019	0.309
Donor type: DBD	0.008	−0.149, 0.165	0.095	0.924
Donor type: DCD	0.152	−0.054, 0.358	1.46	0.145
CYP3A5 expresser	−0.529	−0.845, −0.213	−3.291	0.001
IGF	−0.432	−0.626, −0.238	−4.354	< 0.001
rATG induction	0.005	−0.142, 0.152	0.07	0.944
Standard maintenance immunosuppression	−0.049	−0.265, 0.167	−0.45	0.653
Age (per year increase)	0.012	0.008, 0.016	5.591	< 0.001

Abbreviations: CI, confidence interval, CYP, cytochrome P450; DBD, donation after brain death; DCD, donation after cardiac death; IGF, Immediate graft function; POD, post‐operative day; rATG, rabbit antithymocyte globulin.

## 4 Discussion

We found that kidney transplant recipients with non‐IGF had a higher tacrolimus C/D compared to those with IGF. The impact of early graft dysfunction on tacrolimus C/D differed in CYP3A5 expressers and nonexpressers. In CYP3A5 nonexpressers, patients with non‐IGF experienced a 73% higher tacrolimus C/D when compared to CYP3A5 nonexpressers with IGF. The tacrolimus C/D was only 16% higher in CYP3A5 expressers with non‐IGF versus IGF. This effect was further demonstrated in our regression analysis where both CYP3A5 expression and IGF were significantly associated with the tacrolimus C/D.

A prospective observational study previously evaluated the impact of graft function and CYP3A5 expression on tacrolimus concentrations [8]. Their study observed a 33.6% higher tacrolimus trough concentration in kidney transplant patients with DGF compared to those without DGF. In our study, patients with non‐IGF had a 54% higher tacrolimus C/D compared to those with IGF. Several important differences may have contributed to this discrepancy. First, their study defined DGF as the requirement for dialysis within the first 7 days after transplantation, whereas all remaining recipients were categorized as having IGF. In contrast, our study used a broader definition of non‐IGF that included both recipients meeting the conventional definition of DGF and those with impaired allograft function who did not require dialysis. Consequently, our findings suggest that the impact of graft function on tacrolimus exposure may extend beyond dialysis‐requiring DGF to encompass a broader spectrum of early graft dysfunction. This difference in graft function definitions should be considered when comparing our results with prior studies.

Additionally, tacrolimus concentrations in the previous study were evaluated beginning on POD 1 and were not dose‐normalized, although all patients received standardized dosing. In contrast, we normalized tacrolimus concentrations to dose, allowing evaluation regardless of whether patients received standard dosing or *CYP3A5* genotype‐guided dosing. Although the difference in tacrolimus trough concentrations observed on POD 1 diminished after subsequent dose adjustments, patients with DGF ultimately required lower tacrolimus doses to achieve target trough concentrations in the previous study. Taken together, these findings support the concept that early graft function may be an important factor to consider when individualizing tacrolimus dosing after kidney transplantation.

Although the mechanism of renohepatic crosstalk is not fully understood, the effects of acute kidney dysfunction or AKI on hepatic function have been reported by several studies. A study investigated effects of renal failure on hepatic metabolism and intestinal extraction of tacrolimus in rats, and tacrolimus exposure was significantly increased in rats with renal failure [13]. Notably, the study found that the absorption rate of tacrolimus from the intestine also increased in rats with renal failure indicating that renal dysfunction may alter numerous absorptive and metabolic processes leading to increased exposure. Mechanistically, animal studies indicate that the effects of kidney dysfunction on volume overload, altered metabolism, metabolic acidosis, increased toxins from uremia, and increased inflammatory cytokine production may cause liver dysfunction. The expression of CYP enzymes that accounts for at least 75% of the body's total drug metabolism may be altered due to uremic toxins and proinflammatory cytokines that are produced during AKI [14, 15]. This may explain the effects of early graft dysfunction on tacrolimus exposure seen in this study. These findings are consistent with prior evidence that acute kidney dysfunction can reduce hepatic CYP3A activity. However, because this was an observational study, we cannot exclude the possibility that tacrolimus exposure also contributes to impaired allograft function.

Our findings have important clinical implications. Although a causal relationship between supratherapeutic tacrolimus levels and early graft dysfunction remains under investigation, tacrolimus is a nephrotoxic agent and exposure to supratherapeutic levels may lead to worsening graft function in those already experiencing early graft dysfunction. Based on the results of this study, an empiric tacrolimus dose reduction may be warranted for adult kidney transplant recipients receiving kidneys with characteristics associated with increased risk of early graft dysfunction, especially in CYP3A5 nonexpressers. Donor factors associated with early graft dysfunction, including donor AKI, donation after circulatory death, prolonged ischemic time, and adverse procurement biopsy findings, are generally available before transplantation and could potentially be incorporated into future dosing strategies [16]. However, prospective studies are needed to validate this approach before routine clinical implementation.

This study has several limitations. The retrospective design limited our ability to determine whether a causal relationship exists between high tacrolimus exposure and early graft dysfunction. This study included patients who received different tacrolimus dosing protocols (weight‐based and genotype‐guided). However, using tacrolimus C/D as the primary outcome should minimize the impact of multiple dosing strategies on tacrolimus exposure. POD 2 was intentionally selected to assess tacrolimus exposure before dose adjustments were routinely made; however, this approach does not capture changes in tacrolimus exposure that may occur later in the post‐transplant course as graft function evolves. Lastly, detailed donor characteristics associated with early graft dysfunction, including donor AKI, terminal donor kidney function, procurement biopsy findings, kidney donor profile index, and ischemia times were not available for analysis and therefore could not be evaluated as potential confounders.

Tacrolimus is metabolized by CYP3A4 and CYP3A5 in the liver and intestine. Our study design accounted for differences in CYP3A5, but we were unable to account for differences in CYP3A4 expression. The ratio of CYP3A4 to CYP3A5 metabolism varies by substrate, but the functional effects of *CYP3A4* genotype on tacrolimus exposure are inconclusive [17]. In vitro studies vary in their reports of whether or not *CYP3A4* variants have functional significance in tacrolimus metabolism, but the majority of studies indicate that their clinical significance is low [17]. Although most medications that are known to induce or inhibit tacrolimus metabolism were accounted for in our inclusion criteria, prednisone may decrease the blood concentration of tacrolimus [18]. However, regardless of whether the patient was receiving a standard or steroid‐avoidant immunosuppression protocol, all patients received steroids on POD 2, and the effect of prednisone on tacrolimus exposure was likely small and not clinically relevant.

## 5 Conclusions

Early graft dysfunction in adult kidney transplant recipients was associated with increased tacrolimus exposure. Our findings suggest that CYP3A5 nonexpressers with early graft dysfunction may require lower initial IR‐Tac doses to reduce the risk of supratherapeutic exposure. Although the observed effect size suggests that an approximately 30% to 40% dose reduction could be explored as a potential dosing strategy, this estimate should be interpreted cautiously given the single‐center design and center‐specific dosing practices. As such, these findings should be considered hypothesis‐generating and warrant further investigation. The combined influence of early graft function and *CYP3A5* genotype on tacrolimus exposure highlights a potential opportunity for more individualized dosing early after kidney transplantation. Further multicenter studies are needed to validate these findings and determine whether incorporating early graft function into tacrolimus dosing algorithms improves clinical outcomes.

## Author Contributions

**Meghan Schuff Zimmerman:** writing – original draft, conceptualization, methodology, data curation, project administration, visualization, validation, investigation, writing – review and editing, formal analysis. **Amy L. Pasternak:** conceptualization, investigation, methodology, validation, data curation, formal analysis, visualization, project administration, writing – review and editing. **Snehal Patil:** software, data curation, writing – review and editing, resources. **Sebastian Zöllner:** writing – review and editing, software, data curation, resources. **Jeong M. Park:** conceptualization, investigation, writing – review and editing, visualization, validation, methodology, project administration, resources, supervision, data curation.

## Funding

The authors have nothing to report.

## Conflicts of Interest

Dr. Park is a member of the Pharmacotherapy editorial board. All other authors declare no conflicts of interest.

## Data Availability

The data supporting the findings of this study are available from the corresponding author upon reasonable request. Public sharing of the data is restricted because the dataset contains sensitive participant information and is subject to institutional ethical and privacy requirements.
